# Dental follicle stem cells in bone regeneration on titanium implants

**DOI:** 10.1186/s12896-015-0229-6

**Published:** 2015-12-30

**Authors:** Ondine Lucaciu, Olga Soriţău, Dan Gheban, Dan Rus Ciuca, Oana Virtic, Adriana Vulpoi, Noemi Dirzu, Radu Câmpian, Grigore Băciuţ, Catalin Popa, Simion Simon, Petru Berce, Mihaela Băciuţ, Bogdan Crisan

**Affiliations:** Department of Oral Rehabilitation, “Iuliu Haţieganu” University of Medicine and Pharmacy Cluj-Napoca, 15 Victor Babeș Street, 400012 Cluj-Napoca, Cluj Romania; “Ion Chiricuţă” Oncological Institute Cluj-Napoca, 34-36 Republicii Street, 400015 Cluj-Napoca, Cluj Romania; Department of Anatomic Pathology, “Iuliu Haţieganu” University of Medicine and Pharmacy Cluj Napoca, 1-3 Clinicilor Street, 400006 Cluj Napoca, Cluj Romania; Department of Pathological Anatomy, “Iuliu Haţieganu” University of Medicine and Pharmacy Cluj-Napoca, 8 Victor Babeș Street, 400012 Cluj-Napoca, Cluj Romania; Department of Functional Genomics, Biomedicine and Translational Medicine, “Iuliu Haţieganu” University of Medicine and Pharmacy Cluj-Napoca, 8 Victor Babeș Street, 400012 Cluj-Napoca, Cluj Romania; Faculty of Physics & Institute of Interdisciplinary Research in Bio-Nano-Sciences, Babes-Bolyai University, Cluj-Napoca, Romania; Technical University, Cluj-Napoca, Romania; Department of Cranio-Maxillofacial Surgery, Dental Implantology, “Iuliu Haţieganu” University of Medicine and Pharmacy Cluj-Napoca, 37 Cardinal Iuliu Hossu Street, 400029 Cluj-Napoca, Cluj Romania

**Keywords:** Dental follicle stem cells, Bone regeneration, Bone regeneration on titanium implants, Impacted canine, Impacted molar, Osteogenic markers

## Abstract

**Background:**

We aimed to demonstrate that DF stem cells from impacted molars and canines can be used to improve bone regeneration on titanium implants surfaces. This study highlights the presence of stem cells in DF, their potential to adhere and differentiate into osteoblasts on different types of titanium surfaces.

**Results:**

Isolated cells from the harvested DF tissue from impacted canine/molars, expressed stem cells markers. Differentiation into bone cells was induced in presence or absence of BMP-2 and TGFβ1. The presence of growth factors until 28 days in medium maintained the cells in an earlier stage of differentiation with a lower level of specific bone proteins and a higher expression of alkaline phosphatase (ALP). Influence of titanium implants with different bioactive coatings, hydroxyapatite (TiHA) and with silicatitanate (TiSiO_2_), and porous Ti6Al7Nb implants as control (TiCtrl), was studied in terms of cell adhesion and viability. Ti HA implants proved to be more favorable for adhesion and proliferation of DF stem cells in first days of cultivation. The influence of titanium coatings and osteogenic differentiation mediums with or without growth factors were evaluated. Additional BMP-2 in the medium did not allow DF stem cells to develop a more mature phenotype, leaving them in a pre-osteogenic stage. The best sustained mineralization process evaluated by immuno-cytochemical staining, scanning electron microscopy and Ca^2+^ quantification was observed for TiHA implants with a higher expression of ALP, collagen and Ca^2+^ deposition. Long term culturing (70 days) on titanium surfaces of DF stem cells in standard medium without soluble osteogenic inducers, indicated that HA coating is more favorable, with the acquisition of a more mature osteoblastic phenotype as shown by immunocytochemical staining. These findings demonstrated that even in absence of exogenous osteogenic factors, TiHA implants and in a lesser extent TiCtrl and TiSiO_2_ implants can induce and sustain osteogenic differentiation of DF stem cells, by their chemical and topographical properties.

**Conclusions:**

Our research demonstrated that DF stem cells have a spontaneous tendency for osteogenic differentiation and can be used for improving bone regeneration on titanium implants surfaces.

**Electronic supplementary material:**

The online version of this article (doi:10.1186/s12896-015-0229-6) contains supplementary material, which is available to authorized users.

## Background

Recently, mesenchymal stem cells (MSC) were proven to be present in dental tissues, including dental pulp, periodontal ligament and dental follicle. Dental stem cells are multipotent mesenchymal stem cells that raised new found enthusiasm among the researchers because of their easy accessibility, high quality and the fact that their usage does not involve the same ethical concerns and controversies as embryonic stem cells do.

Dental stem cells are post-natal stem cell populations that have mesenchymal stem cell like qualities, including the capacity for self-renewal and multilineage differentiation potential. These cells are derived from the neural crest, and thus have a different origin from bone-marrow derived mesenchymal stem cells, which are derived from mesoderm [1].

The dental follicle (DF), one of the multipotent tissues, is a fibrous ectomesenchymal tissue sac that surrounds the unerupted tooth [2] and regulates the osteoclastogenesis and osteogenesis needed for tooth eruption [3]. Dental follicle stem cells are the origin of the periodontium, including cementum, periodontal ligament and alveolar bone [4, 5] and this developmental cascade confirms the existence of stem cells in the dental follicle. Cells from the dental follicle are able to differentiate toward cementoblasts, fibroblasts and osteoblasts (elaborating the alveolar bone) [6]. Precursor cells isolated from dental follicle express STRO-1, Oct3/4, Sox-2, Nanog, Notch1 and nestin (markers of multipotential mesenchymal progenitor cells) [7, 8] and are negative for markers of hematopoietic lineage CD34 and CD117, and positive for CD44, CD29, CD 90 and CD105, indicating that those cells are mesenchymal cells [9]. Dental follicle-derived stem cells have excellent proliferation rates and under specific culture conditions have the capacity to differentiate in multiple lineages cell types such as osteoblasts, cementoblasts, adipocytes and neuron-like cells [1, 10].

Most people have an impacted third molar or canine that does not cause occlusion and usually have the impacted tooth extracted either to avoid inflammation or for orthodontic therapy. Such extracted teeth usually contain dental follicle and are commonly discarded as medical waste. Hence, the dental follicle is a candidate source for isolating stem cells.

Studies in the literature have demonstrated the presence of stem cells in the dental follicle of wisdom teeth [11], but there is no research related to the presence of these cell populations in the canine follicular bag included. Another issue less studied is the use of follicular stem cells in improving bone regeneration on titanium implants surfaces. Current approaches of solving bone defects using alloplastic materials (titanium, ceramics) can involve important limitations, such as weakness of osseointegration in the case of long-term implants, inflammatory response at the implantation sites, biomechanical mismatch. These clinical problems determined further research in this field of implantology, including interventions on implants features in terms of biocompatibility (physicochemical properties and surface bioactivity) and also on behavior of cellular components implicated in osteointegration process.

Given the above background, we aimed to demonstrate that dental follicle stem cells from impacted molars and canines can be used to improve the bone regeneration process on titanium implants surfaces. The study highlights the presence of stem cells in the dental follicle, their potential to differentiate into bone line cells and to adhere and differentiate into osteoblasts on different types of titanium surfaces.

## Results

**Fig. 1:**
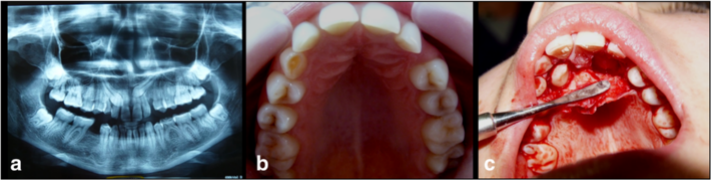
**a** radiological aspect of the two impacted canines. **b** clinical aspect of the two impacted canines. **c** surgery aspect from the removal of the dental follicle surrounding the impacted canine

In this study we present the harvesting technique of the dental follicle from an impacted upper left canine with palatal position. Figure 1a illustrates the radiologic appearance of canine 23 with intra-osseous impactation from which the follicular sac was harvested. Figure 1b shows the clinical appearance of impacted left and right upper canines. After disinfection of the surgical site under local anesthesia, we have performed a palatal incision along the cervical lines from tooth 15 to 25, using a scalpel with a no.15 blade. We reflected the muco-periostal palatal flap, preserving the nasopalatine nerve. We removed the bone covering the crown of the impacted left canine. Immediately after exposure of the impacted left canine, we harvested the follicular sac that was surrounding his crown. Dental follicular tissue was harvested using a scalpel with a no.15 blade. The crowns of both impacted canines were exposed but no orthodontic brackets for traction of the teeth were placed (Fig. 1c). The area was then irrigated with saline solution and the flap was closed with interrupted sutures.

### Histological analysis of the harvested tissues

**Fig. 2:**
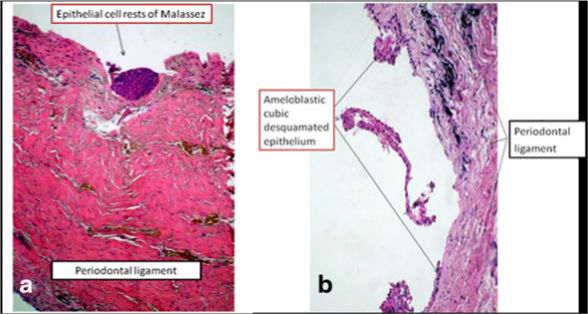
Histological aspect of the canine dental follicle harvested tissue. **a.** Malassez epitelial rest and **b.** ameloblastic epithelia on periodontal ligament

Histological analysis of the harvested tissue from impacted third molar and canine reveals the typical aspect of dental follicle with ameloblastic cubic epithelium, Malassez epithelium. First image (Fig. 2a) presents on the top of a vasculo-connective tissue forming the periodontal ligament, a small cluster of epithelial residual cells from Hertwig’s epithelial root sheath (Epithelial cell rests of Malassez). These cells are remains from epithelial cells located at cervical loop of the enamel organ in a developing tooth. They initiate the formation of dentin in the root of a tooth by causing the differentiation of odontoblasts from the dental papilla. Second image (Fig. 2b) presents a fragment of detached ameloblastic epithelia part from a scratched dental follicle.

### Establishment of cell culture from the dental follicle

**Fig. 3:**
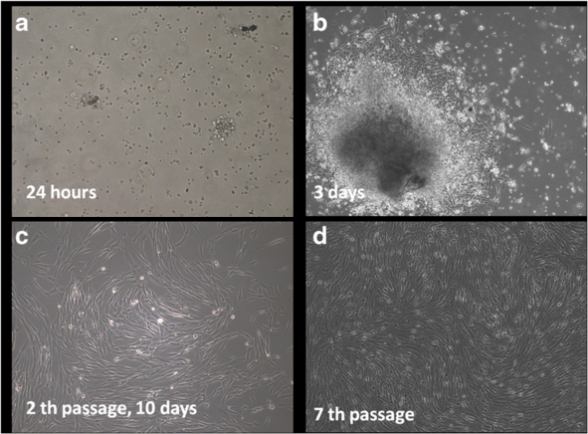
Morphological aspects of isolated cells from dental follicle in contrast-phase microscopy. (**a**) after 24 h rounded shape cells adhere to plastic surface; (**b**) migrated cells after 3 days from explants presented a fibroblast-like appearance; (**c**) morphologically homogeneous fibroblast-like cell population was observed at 2th passage; (**d**) cells maintained their high proliferation rate at passage 7th (magnification ×100)

After initiation of cell adhesion in first 24 h, cell proliferation was intensive, and after achieving of subconfluence within 3 days, the first passage was made. Morphological aspect of the cells was a fibroblast type, spindle-like shape (Fig. 3c). These cells maintained a high proliferation rate even at advanced passages without modification of morphological aspect (Fig. 3d). Cells culture needed passages at 2–3 days.

### Immunophenotypic analysis

**Fig. 4:**
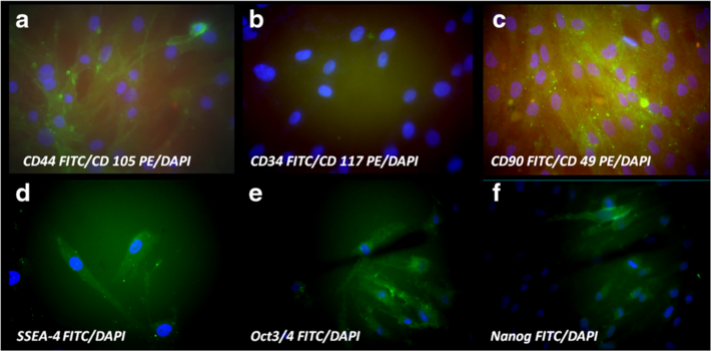
Immunocytochemical staining for characteristic stem cells markers, nuclei were counterstained with DAPI. (**a**) strong expression for CD 44 FITC and weak positivity for CD 105 PE; (**b**) negative staining for CD 34 FITC and CD 117 PE; (**c**) strong positivity for CD 90 FITC, weak expression of CD 49 PE; (**d**) strong positivity for early embryonic antigen SSEA-4 FITC; (**e**) the self-renewal embryonic proteins Oct3/4 FITC strong expression and (**f**) Nanog FITC positive in some cells (magnification ×400)

Immunocytochemical staining performed for isolated cells at third passage has shown strong positivity for specific markers of mesenchymal stem cells such as: SSEA-4, Oct3/4, Nanog, CD44, D90, and weak positivity for CD105 and CD49e. Cells did not express CD 117 and CD34 (Fig. 4).

**Fig. 5:**
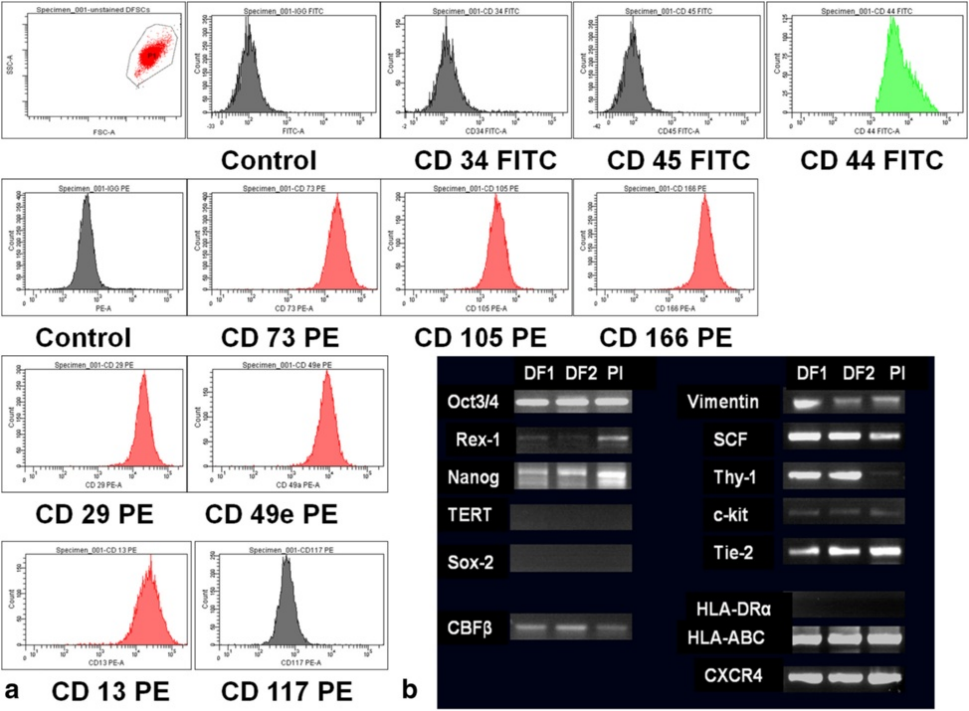
**a** Cell surface stem cell antigens analysed by flowcytometry: positive expression for CD44, CD73, CD105, CD29, CD49e and CD166 and lack of expression of CD34, CD45 and CD 117 indicate a mesenchymal stem cell phenotype. **b** RNA molecules expression in dental follicle (DF1, DF2) and placental (Pl) mesenchymal stem cells: strong expression for Oct3/4, Nanog, SCF,CXCR4, Thy-1 and Tie-2 and HLA-ABC, and absence of HLA-DR, Sox-2,c-kit, and TERT expression. Vimentin and Rex-1 have a weaker expression and was different for DF1 and DF2

Similar results were obtained by flowcytometry analysis: negative staining for CD34 FITC, CD45 FITC and CD117 PE and positivity for CD44 FITC, CD73 PE, CD105 PE, CD29 PE, CD49e PE, CD166 PE (Fig. 5a).

### RT-PCR analysis for stem cells gene expression

RT-PCR analysis performed in comparison with placental-derived mesenchymal stem cells, confirmed the phenotypic analysis. Isolated cells from dental follicle showed strong expression of Oct3/4, CXCR4, SCF (stem cell factor), CBFβ, Thy-1,Tie-2, vimentin, nanog, and HLA-ABC. These cells didn’t express hTERT, Sox-2, c-kit and HLA-DRα (Fig. 5b).

### Osteogenic differentiation of dental follicle stem cells in vitro

#### The advantage of simple osteogenic medium vs. complex osteogenic medium in inducing bone specific proteins

**Fig. 6:**
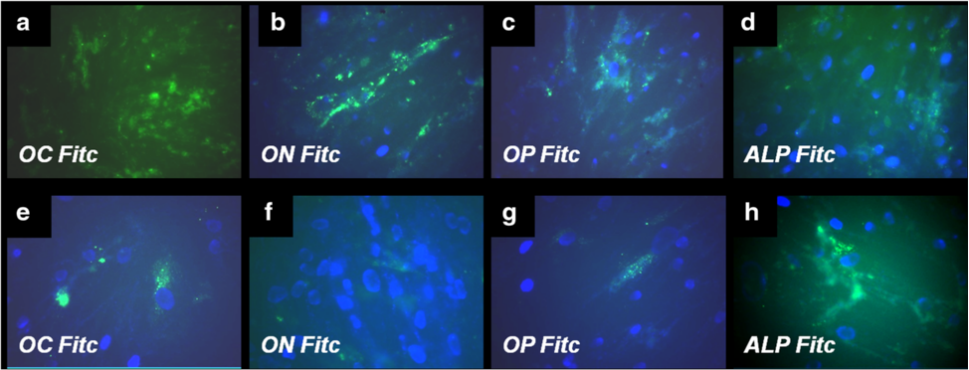
Fluorescence images of immunocytochemical staining of DF stem cells induced for osteogenic differentiation using simple osteogenic medium in *upper panel*: (**a**) osteocalcin (OC FITC), (**b**) osteonectine (ON FITC), (**c**) osteopontin (OP FITC) and (**d**) alkaline phosphatase (ALP FITC). In the *lower panel* are illustrated the induced expression of same osteogenic markers when DF stem cells were cultivated in presence of complex osteogenic medium: (**e**) OC FITC, (**f**) ON-FITC, (**g**) OP-FITC and (**h**) ALP-FITC. Nuclei were counterstained with DAPI (Magnification ×400)

A comparative study was carried to determine the influence of culture medium composition on osteogenic differentiation of DF stem cells. The simple osteogenic medium consisting of ascorbic acid, β glicero-phosphate and dexamethazone induced a more stronger expression of non-collagenous proteins osteocalcin (OC), osteonectine (ON), osteopontin (OP) and alkaline phosphatase (ALP) as shown by immunocytochemical staining of cells after 3 weeks of cultivation. A certain difference is observed between OC and OS medium, the cells cultivated with OS medium expressed in a higher extent bone specific non-collagenous proteins OP, ON and OC (Fig. 6a, b, c). Instead, for cells cultivated with OC medium we noticed a more intense staining for ALP (Fig. 6h). Cells exhibited a particular patch distribution of bone markers present both in intracellular and extracellular space, related with formation of the novo bone matrix. We also compared the DF stem cells cultivated in standard stem cell medium for 3 weeks in terms of osteopontin and alkaline phosphatase expression. A weak expression is observed in these images for ALP and negative staining for OP as shown in Additional file 1: Figure S5.

The complex osteogenic medium, that additionally contains growth factors (BMP2 and TGFβ1) has proved less favorable in bone specific proteins expression, but with a more intensive expression of alkaline phosphatase (ALP).

### Cultivation on titan implants

#### Cell adhesion and viability of DF stem cells in short term cultures on titanium implants

**Fig. 7:**
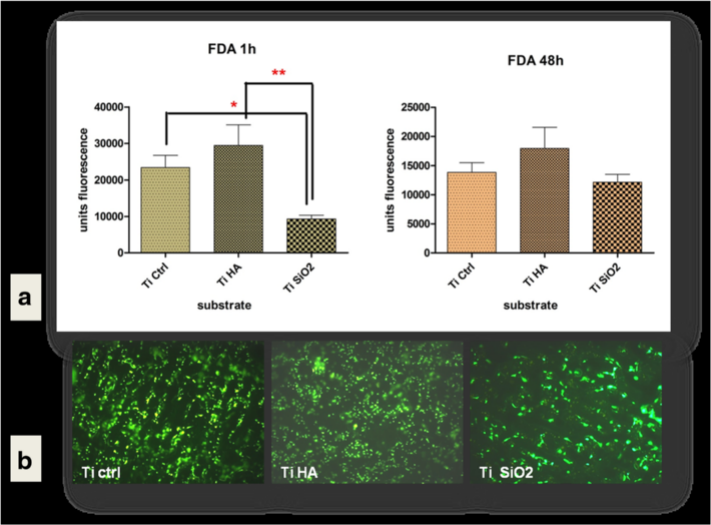
**a** DF stem cells adhesion on titanium implants after 1 h and cell viability at 48 h evaluated by fluorescein diacetate (FDA) test (area scan) (**b**) Fluorescence microscopy images of FDA stained DF stem cells cultivated 7 days on titanium surfaces in standard stem cells medium (Legend: TiCtrl- Ti6Al7Nb alloy porous titanium, TiHA-titanium infiltrated with hydroxyapatite, TiSiO_2_-titanium infiltrated with silicatitanate) (magnification ×100)

We investigated the behavior of DF stem cells cultivated on surfaces of titanium implants, in order to lay the foundation for finding a new way to induce bone regeneration on the titanium implant surface. The adhesion process was evaluated after 1 h of cultivation of DF stem cells in standard stem cells medium on three types of titanium implants (TiCtrl, TiHA and TiSiO_2_) using the fluorescein diacetate test (FDA). The highest fluorescence values were found for TiHA and Ti Ctrl implants with statistically different values comparing with TiSiO_2_ implants (statistical analysis was performed using One-way analysis of variance; *p* value < 0.001). The most favorable substrate was proved to be titanium implants infiltrated with HA, especially in the first hour of cell adhesion process. The differences were statistically significant at 1 h after seeding the cells. At 48 h and at 7 days of cultivation the HA infiltrated titanium implants preserved the advantages for cell proliferation, but the differences were not statistically significant (Fig. 7). Microscopical analysis of FDA stained DF stem cells confirmed the increased number of cells after 48 h and 7 days for DF stem cells cultivated on Ti Ctrl and TiHA implants (Additional file 2: Figure S2). Cell viability and subsequent cell proliferation were evaluated by an additional viability test (Alamar blue) in two conditions: (1) in standard stem cells medium and (2) in a comparative study between stem medium and differentiation medium OS and OC. Alamar test revealed as FDA test that in the first day of cultivation the Ti HA offers slightly increased DF stem cells adhesion, but there are no differences between implants after 4 and 12 days in terms of viability and proliferation (Additional file 3: Figure S3). These findings are strengthened by the results obtained for the cells cultivated with stem cell medium and osteogenic medium for 4 and 12 days. The differences appeared between stem cell medium and osteogenic differentiation medium, as inducing the osteogenic differentiation had caused, as expected, a decrease in cell numbers after 4 and 12 days of cultivation (Additional file 4: Figure S4).

#### The influence of implants surface and culture medium on BMP-2 and osteopontin expression during osteogenic differentiation of DF stem cells

We evaluate the influence of titanium implants chemistry and topography in combination with differentiation medium on DF stem cells osteogenic differentiation. BMP-2 is implicated in stem cells differentiation into osteoprogenitor cells. BMP-2 de novo secretion was determined by ELISA method, in conditioned media at 8, 14 and 21 days of DF stem cells culturing on titanium implants. The TiCtrl implants induced the highest BMP-2 secretion at 14 days in OS medium. A very similar behavior was observed for all types of control titanium implants cultivation mediums, by achieving a peak at 14 days and decreased values at 21 days.

**Fig. 8:**
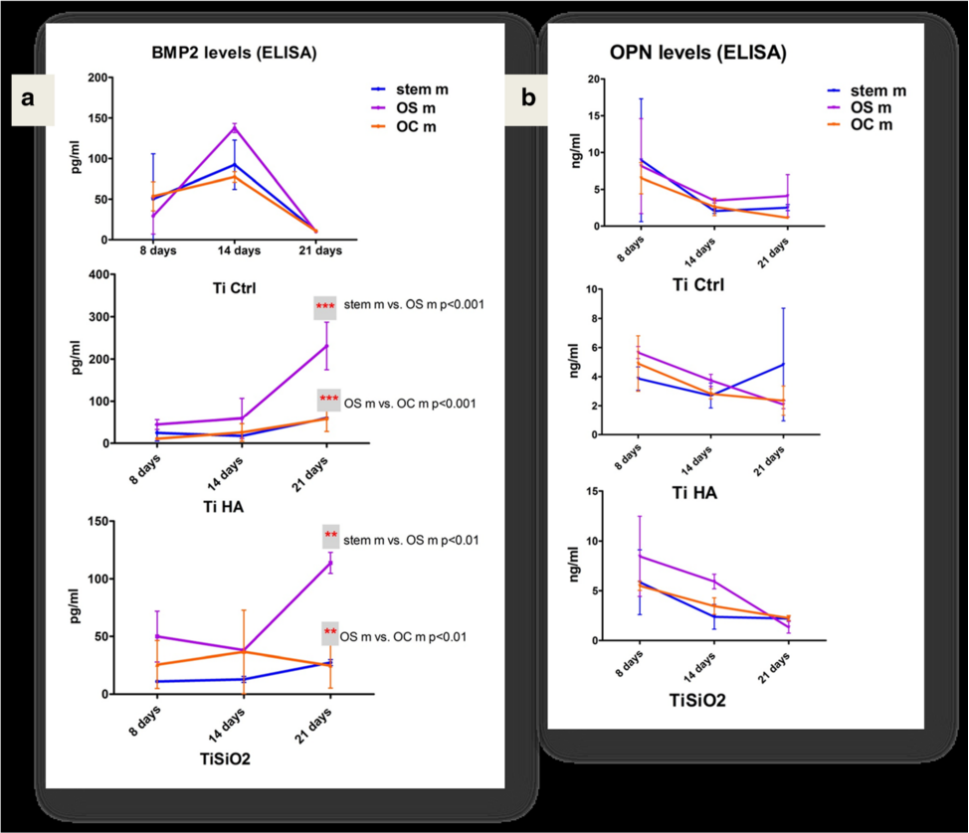
**a** BMP-2 expression in cell culture medium of DF stem cells cultivated on titanium implants in relation with differentiation medium: standard stem cell medium (*blue*), simple osteogenic medium (OS) (*purple*) and complex osteogenic medium (OC) (*orange*). **b** Osteopontin (OPN) values obtained in cell culture medium in the same conditions of DF stem cell cultures. (Legend: TiCtrl- Ti6Al7Nb alloy porous titanium, TiHA-titanium infiltrated with hydroxyapatite, TiSiO_2_-titanium infiltrated with silicatitanate; stem m-stem cell medium, OS m- simple osteogenic medium; OC m- complex osteogenic medium)

For TiHA implants the BMP-2 values were in a continuous increase for all types of mediums. We observed the maximum values in the samples with TiHA in OS medium (purple line) at 21 days, with values almost 1.5 times larger compared to the highest levels obtained for TiCtrl and TiSiO_2_ samples. For stem cell (blue line) and OC medium (orange line) BMP-2 levels at 21 days were very close to the other measurements from the days 8 and 14. We obtained statistical differences only at 21 day between values for OS medium and stem cell medium, respectively OC medium (Fig. 8a).

For TiSiO_2_ the highest BMP-2 levels were recorded at 21 days of cultivation in OS medium, while in stem cell BMP-2 values increased slightly and in the OC medium the BMP-2 values decreased at 21 days. Differences in inducing BMP-2 secretion were observed between OS, stem cell and OC mediums at 21 days of cultivation. Statistical analysis was carried out using two-way ANOVA Bonferroni post test, and revealed statistical significant differences between the mediums for TiHA and TiCtrl implants (*p* value < 0.001) (Fig. 8a).

Osteopontin expression evaluated by ELISA test, showed a maximum value in first 8 days of cultivation on titanium implants, especially for TiCtrl and TiSiO_2_. The levels of osteopontin progressivelly decreased toward 21 days of cultivation. At 14 days the OS medium sustained the highest osteopontin expression on TiSiO_2_ implants (Fig. 8b).

#### The mineralization process is in relationship with surface bioactivity of titanium implants and differentiation cell culture medium

**Fig. 9:**
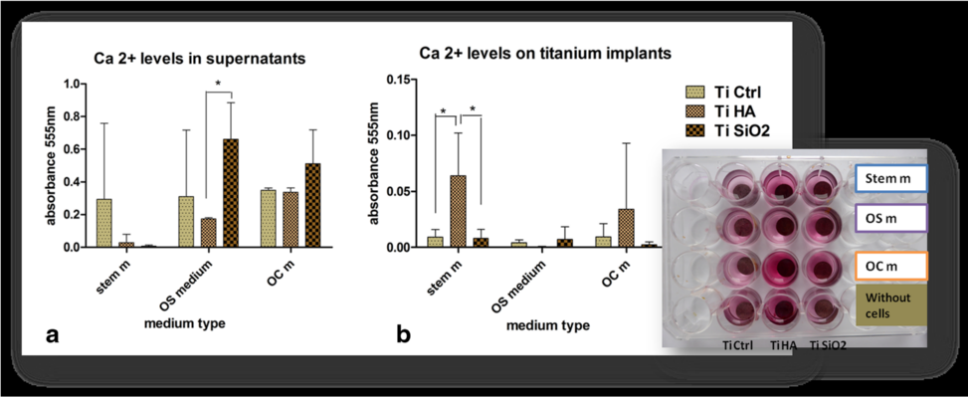
Calcium quantification by the alizarin red method. **a** Ca^2+^ levels in supernatants harversted after 21 days, from the DF stem cell cultures on Ti Ctrl, Ti HA, Ti SiO_2_ implants using standard stem medium, OS and OC mediums (**b**) alizarin red staining of Ca^2+^ deposition at the surface of Ti Ctrl, Ti HA, Ti SiO_2_ implants, in same the conditions of cultivation for 21 days. (Legend: TiCtrl- Ti6Al7Nb alloy porous titanium, TiHA-titanium infiltrated with hydroxyapatite, TiSiO_2_-titanium infiltrated with silicatitanate; stem m-stem cell medium, OS m- simple osteogenic medium; OC m- complex osteogenic medium)

The mineralisation process was assessed by determing the Ca^2+^ levels from the supernatants obtained after 21 days of culturing DF stem cells on titanium implants, in stem cell medium, simple and complex osteogenic medium. We also evaluated the Ca^2+^ content at the surface of the titanium implants after 21 days of culture. The results are illustrated in Fig. 9. High levels of Ca^2+^ can be observed in the supernatants obtained from cells cultured in the presence of simple and complex osteogenic medium especially on TiSiO_2._ Supernatants obtained from cells cultured on TiCtrl implants revealed increased Ca^2+^ values regardless of the type of culturing medium (Fig. 9a). Titanium surfaces coated with hydroxyapatite in the presence of stem cell medium and complex osteogenic medium proved to be the most favorable in terms of Ca^2+^ deposition on the implants’ surface (Fig. 9b). Titanium implants maintained for 21 days in stem cell medium without cells revealed also positivity for alizarin staining, more intensively for implants treated with hydroxyapatite. Those implants were kept in the same conditions with implants seeded with cells and had time to adsorb on the surface of implants the existing calcium in basal medium and FBS.

**Fig. 10:**
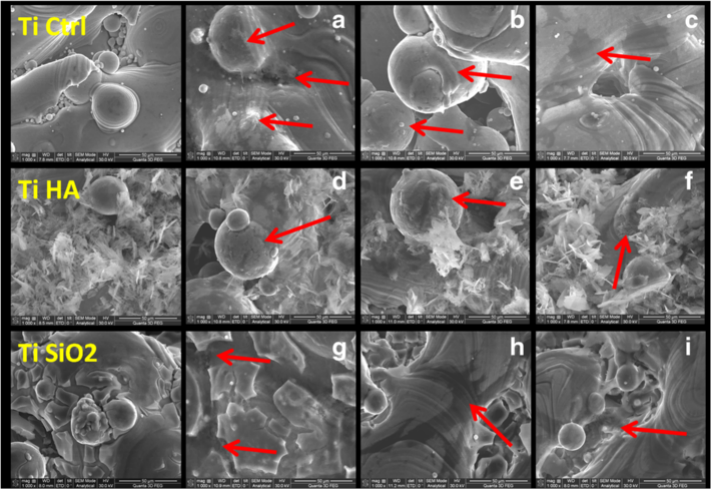
SEM images of titanium implants seeded with DF stem cells. *Left panel* -TiCtrl, TiHA, TiSiO_2_ implants without cells (**a**) TiCtrl with stem cell medium (*arrows* indicate matrix deposition) (**b**) TiCtrl with simple osteogenic medium (*arrow*-cell surrounded by bone matrix) (**c**) TiCtrl with complex osteogenic medium (*arrow*-flattened cell with numerous extensions) (**d**) TiHA with stem cell medium (*arrow*-strong matrix deposition) (**e**) TiHA with simple osteogenic medium (*arrow*-cell surrounded by bone matrix) (**f**) TiHA with complex osteogenic medium (**g**) TiSiO_2_ with stem cell medium (*arrows* indicate matrix deposition inside of grooves) (**h**) TiSiO_2_ with simple osteogenic medium (*arrow*-large flattened cell with numerous extensions) (**i**) TiSiO_2_ with complex osteogenic medium (*arrow*- embedded cells in matrix) (magnification ×1000)

Scanning electron microscopy (SEM) has brought additional information regarding the changes of implants’ surface and cell behavior for long term cultivation. After 21 days of cultivation on titanium implants, we observed de novo matrix deposition especially on TiHA and TiCtrl implants, in cultures with stem cell medium and OS differentiation medium (Fig. 10a, b, d, e). The smooth surface of TiSiO_2_ implants has been changed in time by cultivation in the given microenviroment conditions with appearance of grooves that allowed the deposition of the newly formed matrix. For TiSiO_2_ samples, the most favorable medium seems to be the complex osteogenic medium (Fig. 10g, h, i).

#### Increased osteogenic differentiation of DF stem cells cultivated on hydroxyapatite coated titanium implants in standard stem cells medium

**Fig. 11:**
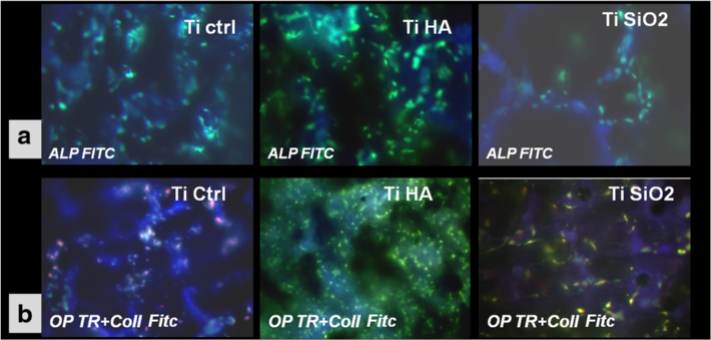
**a** Fluorescence images of immunocytochemical staining for alkaline phosphatase (ALP FITC) expression after 70 days of cultivation of DF stem cells on Ti implants surface (TiCtrl-porous Ti6Al7Nb titanium, TiHA-titanium infiltrated with hydroxyapatite, TiSiO_2_-titanium infiltrated with silicatitanate) in standard stem cells medium (magnification ×200). **b** Immunocytochemical staining for osteopontin (OP marked with *texas red*) and collagen (Coll marked with FITC) of DF stem cells cultivated in standard conditions on Ti implants surface (TiCtrl-Ti6Al7Nb porous titanium, TiHA-titanium infiltrated with hydroxyapatite, TiSiO2-titanium infiltrated with silicatitanate) (magnification ×200)

We hypothesized that osteogenic DF stem cell differentiation could be initiated in absence of main soluble osteogenic factors used in standard protocols, such as ascorbic acid, β-glycero-phosphate, dexamethazone, and growth factors. We used in these experiments as osteogenic inductors only the titanium surfaces with or without bioactive coatings. The expression of specific osteogenic markers collagen 1A (Coll), osteopontin (OP) and alkaline phosphatase (ALP) were determined after 70 days of cultivation of cells on the three types of titanium implants surface. The first finding regarding immunocytochemical staining was the increased number of cells observed on TiHa implants surface with a strong expression of alkaline phosphatase as shown in Fig. 11. The proliferation rate was lower for TiCtrl and TiSiO_2_, but the cells expressed also strong ALP positivity.

Collagen expression was higher for cells cultivated on titanium implants with bioactive coatings (stronger for TiHA, and less strong for TiSiO_2_). Staining cells for osteopontin revealed that control titanium and TiSiO_2_ induced the greatest expression of this non-collagenous protein in DF stem cells (Fig. 11).

## Discussions

The goal of our research was to prove that dental follicle stem cells are a valuable cell source in improving bone regeneration on titanium implants surfaces. The dental follicle harvesting procedure is easy to undertake since it can be performed in relation to several surgical operations, impacted tooth extraction or even less invasive tooth disclosure. The search for alternative sources of mesenchymal stem cells (such as the dental follicle) is of considerable importance because bone marrow aspiration is an invasive procedure [1] and there are significant age-related decreases in the frequency and differentiation potential of bone-marrow-derived MSCs [12]. Compared to other stem cell sources from the oral cavity, as tooth pulp, the dental follicle is a considerably larger tissue [2], is easily accessible and dental follicle stem cells have a higher proliferation capacity than stem cells from the tooth pulp [13].

One innovative aspect of this study is that the follicular sac can be harvested by orthodontic exposure, thus making the odontectomy of the canines not needed for the harvesting. Dental follicular tissue harvesting is easier when removing impacted teeth than in the case of orthodontic exposure of an impacted canine. After removing the impacted molar, the follicular sac can be easily exposed and harvesting can be done using scissors or scalpel with a no.15 blade. In case of impacted canine exposure for orthodontic reasons, harvesting of the dental follicle can be done only be scratching of the follicle sac from the crown of the impacted canine. Only small fragments of follicle sac can be obtained by orthodontic exposure of impacted canine. A correct diagnosis of complete intrabone impacted canine/molar is a precondition of obtaining uncontaminated dental follicle. Partial impacted canine/molar can lead to contamination of the follicular sac, thus compromising the harvesting procedure. This procedure is minimally invasive, and it allows stem cell harvesting during a surgical procedure that was needed anyway. Easy harvesting of the follicular sac when coupled with minimal invasive surgery allows for stem cell harvesting during odontectomies/orthodontic exposure, as well as storage in a stem cell bank, as standard procedure [14].

The dental follicle that we obtained from human impacted molars and canines had the same normal histological structure without pathological alterations as shown in Fig. 4. We demonstrated that the isolated cells by adherence property to plastic surfaces are stem cells with high proliferation rate, which suggests their remarkable capacity of self-renewal and propagation. Reports about the increased proliferation rate and colony-forming capacity of DF stem cells by comparison with dental pulp MSCs, are considering that DF stem cells have more advantages as a stem cells source [2, 13]. Their ability to differentiate into a wide variety of cell types, together with the possibility of obtaining a high number of cells, make them good candidates for the repair and regeneration of a large variety of tissue [2].

In our research, intense positivity for markers as Oct ¾, Nanog, SSEA-4, CD90, CD 44, CD 29 and CD 73 negative staining for CD 34, Cd 117 and CD 45, and low positivity for CD 105 and CD49e, along with the high proliferation rate, suggests an intermediate stage between embryonic and adult stem cells of obtained cells from the human dental follicle. The ability of DF stem cells to differentiate into neuronal cells positive for neurofilaments as shown in Additional file 5: Figure S1 has demonstrated the plasticity of DF stem cells. Similar results of high positivity levels (around 80–100 %) for embryonic stem cells markers SSEA-4, Oct-4, TRA 1-60, TRA 1-81, and a lower percentage for CD 90 and CD 133 were reported and are arguments for DF stem cells pluripotency [15]. Morsczeck et al. [11] and G.T.-J. Huang et al. [16] demonstrated the presence of undifferentiated cells using Nestin and Notch-1 as markers. Ponnaiyan et al. [17] (2014) reviewed the literature and tried to establish a distinct phenotypic fingerprint of each type of dental stem cells, to be more easy to identify and use in regenerative medicine. From this point of view, DF stem cells compared with bone-marrow MSCs expressed higher amounts of IFF-2 transcripts and positivity for vimentine. An important observation in our experiments was the expression of Oct-4, Nanog and Rex-1 (though weak) transcription factors, which are considered to be specific for pluripotent stem cells [18, 19]. Rex-1 is involved in maintaining an undifferentiated state of ESCs and it is also expressed in bone marrow MSCs, but its function in the dental follicle is unclear. As we are talking about a mesenchymal type of stem cells, it was not only expected, but necessary for them to express CD90 (Thy-1) and vimentin [20]. CD90 is an antigen which is present on CD34^+^ cells from human bone marrow, umbilical cord blood and fetal liver. The significance of CD90 expression on MSCs as a possible marker for further osteoblastic differentiation is in debate.

Using RT-PCR, we could identify the expression of SCF and, faintly, of its receptor c-kit. These molecules mostly regulate the proliferation and survival of early hematopoietic cells, but also of germinal cells and melanocytes. The cells don’t seem to express the telomerase transcript (TERT), in agreement with their lack of immortality in culture. This can actually be viewed as a good thing, namely an almost nonexistent risk of malignant tumors after transplantation. DF stem cells we have examined have a complete lack of HLA-DR. This comes not only as a confirmation of their immune privileged status, but as an essential requirement for their definition as MSCs [20]. The lack of expression of HLA-DR offers immunosuppressive properties to these cells whit major impact in clinical transplantation. Lack of rejection of DF stem cells reduces the incidence of graft- versus host disease as shown by Le Blanc [21], Frank MH [22]. These data suggest once more that DF stem cells are a valuable source of cells for tissue engineering. On the other hand, the immunomodulatory properties of DF stem cells by inhibition of cytokine secretion, T and NK cells proliferation, B cell and dentritic cells maturation and activation, make them a very good cell source for cell based therapy of immune and inflammation-related diseases [23, 24].

A very important factor in elucidating the cellular basis of tissue regeneration is determining the multipotential capabilities of stem cells to differentiate into desire target tissue [25]. Odontogenic tissues deriving from neural crest such as DF stem cells, shows typical features of multipotency and are characterized by a high degree of plasticity, with capacity to differentiate in cells lines derived from all three germ layers (muscle cells, osteoblasts, neuronal and glial cells, adipocytes) as d’Aquino et al. demonstrated [15]. An intensively field of investigation have been focused on the role of MSCs in bone formation, and on DF stem cells in particular [2, 26, 27].

In line with the international research carried out, we demonstrated that dental follicle stem cells have the capacity to induce mineralization in vitro and that new bone formation might be possible using DF stem cells. Based on existing information, we studied the optimal cell culture conditions and the biocompatibility of several substrates with DF stem cells induced to differentiate into osteoblasts. Exposure to osteogenic differentiation environment, such as soluble factors (ascorbic acid, β-glycerol phosphate, dexamethazone, and growth factors BMP-2 and TGFβ1) induced osteogenic differentiation of DF stem cells in our experiments. Simple osteogenic medium without growth factors highly increased the expression of osteoblast main non-collagenous proteins (osteopontin, osteonectin, osteocalcin) as shown by immunocytochemical staining. The expression of ALP was increased in complex osteogenic differentiation medium containing BMP-2 and TGFβ1. Although ALP is a membrane marker of all types of stem cells, is also a marker of osteogenic differentiation. In early stage of differentiation (days 5–14) an initial peak of ALP is observed, followed by a gradual decrease. In this stage, collagen type I is deposited in de novo synthesized extracellular matrix. The final stage of osteogenic differentiation (days 14–28) is characterized by high levels of osteocalcin and osteopontin, and deposition of calcium phosphate [28]. Cells’ response to growth factors in complex osteogenic medium observed in our experiments, can be explain by the expression of homeobox DLX3 transcription factor which regulates osteogenic differentiation via a BMP-2-dependent pathway in DF stem cells. The osteogenic differentiation via a BMP-2 dependent pathway is activated in presence of BMP-2 as well as in presence of dexamethasone and insulin, as shown by Viale-Bouroncle et al. (2012). BMP-2 induces the expression of DLX3 and, in turn, DLX3 induce BMP-2 expression. DLX3 maintains cells viability and morphology (by reorganization of actin filaments) and plays an important role in regulation of osteogenic genes and matrix mineralization [29]. This study reported that BMP-2 induced high activity of ALP in DF stem cells, but this ALP activity was supposed to be directly coordinated by DLX3. After overexpression of DLX3 in DF stem cells, a late osteogenic transcription factor *ZBTB 16* is up-regulated, by a RUNX2 independent pathway [30]. In our study the presence of BMP-2 until 28 days in the complex osteogenic medium maintained the cells in an earlier stage of differentiation with a lower level of collagen and non-collagenous proteins and a higher expression of ALP. We can suppose that BMP2 and dexamethasone induced the NOTCH signaling pathway (involved in some cellular processes such as cell proliferation and stem cell niches maintenance) via a negative BMP-2/DLX3 feed-back loop [31].

Due to their high proliferation features and capacity to differentiate into osteoblasts, DF stem cells offer a great potential for the future of clinical dentistry [32]. Lately, the research in the field of bioactive materials aims to develop improved materials with a higher rate of success in the osteointegration process and in inducing bone regeneration. The materials selected for tooth implants should be biocompatible with host tissue, and permit recruitment of endogenous stem cells [33]. Combining MSCs with biomimetic implants (titanium, ceramics, natural and synthetic polymers coated with bioactive molecules) represents an research area that aims to improve surface topography [34] of dental implants, by that improving the osseointegration process. DF stem cells were cultured on a β-tricalcium phosphate (TCP) scaffolds [35], and the conclusion of these studies was that TCP supports osteogenic differentiation in dental follicle stem cells but also that it induces programmed cell death. Recent studies, tested other substrates, biomaterials with certain surface topographical properties (calcium phosphate thin sputter films on nanostructured titanium surfaces) capable to recruit bone marrow MSCs and promote their osteogenic differentiation. Cell adhesion and proliferation, expression of key osteogenic markers were induced on structured titanium with calcium phosphate coating [36].

Similar results were obtained in our study. We used titanium implants as scaffolds for DF stem cells. Different titanium implant surfaces (porous Ti6Al7Nb alloy–as Ti control, hydroxyapatite coated titanium-TiHA and silicatitanate coated titanium-TiSiO_2_) were evaluated for biocompatibility with DF stem cells, using the FDA viability test. The test showed that titanium surfaces treated with HA are more favorable to adhesion and proliferation.

We assessed the influence of titanium surfaces and differentiation medium on DF stem cells osteogenic differentiation by BMP-2 and osteopontin level evaluation. Figure 8 shows that DF stem cells cultivated on titanium coated with hydroxyapatite with simple osteogenic medium, expressed the highest levels of BMP-2 at 21 days after the culture initiation. BMP-2 levels are found an increasing trend from day 8 to day 21 of cultivation in simple osteogenic medium for TiHA and TiSiO_2_ implants_._ The complex osteogenic medium does not induce high levels of BMP-2. Additional BMP-2 does not allow stem cells to maturate into osteogenic cells, leaving them in a pre-osteogenic stage. Based on that, we can conclude that BMP-2 fails to stimulate bone regeneration and osseointegration as also shown by Chaudhari A. et al. [37].

Osteopontin levels reached a maximum value in the first 8 days of cultivation on titanium implants. The osteopontin levels presented a decreasing pattern of values toward day 21. The high levels of osteopontin detected at 8–14 days of culture, indicate early stages of bone matrix formation. In this early stage of bone formation the secretion of bone proteins builds up a dense network of matrix proteins that helps cells anchor to the titanium surfaces [38]. The generated osteopontin will bind strongly to the available crystals and determine the mineralization of the extracellular matrix. Alizarin red staining (Fig. 9) for Ca^2+^ quantification indicates the highest levels of Ca for the implants treated with hydroxyapatite. Titanium surfaces that were treated with SiO_2_ present high levels of Ca^2+^ in the supernatant and not on the surface of the implants, this can be explained by the release of silicic acid in culture medium [39, 40] leading to intense Alizarin Red positivity.

The immunocytochemical staining revealed that the highest numbers of cells were to be found on TiHA implants with a high expression of alkaline phosphatase and collagen. SEM has also correlated the previous finding. The electron microscopy images indicate the presence of cells on the surface of TiHA with matrix deposition as shown in Fig. 10. In the authors’ opinion, coating titanium implants with hydroxyapatite determines a higher cell adhesion rate than when compared to control titanium surfaces or SiO_2_ treated surfaces. Our research is in line with the findings of Vamze J et al. (2015) who concluded that pure hydroxyapatite stimulates the potential of bone regeneration [41].

Long term culturing (70 days) of DF stem cells in standard medium without soluble osteogenic inducers on Ti6Al7Nb alloy,TiHA and TiSiO_2_ surfaces, induced in all samples the expression of specific osteoblasts markers: ALP, osteopontin and collagen. Hydroxyapatite coating of titanium implants has been proved more favorable, with the acquisition of a more mature osteoblastic phenotype as shown by immunocytochemical staining (increased ALP expression, weak positivity for osteopontin and increase expression of collagen). These findings demonstrated that even in absence of exogenous osteogenic factors, TiHA implants and in a lesser extent TiCtrl and TiSiO_2_ implants can induce and sustain osteogenic differentiation of DF stem cells, by their chemical and topographical properties.

The presented research aims to lay the groundwork for further clinical procedures of harvesting and storage of DF cells in stem cells banks.

## Conclusion

Dental follicle stem cells have been easily obtained from clinically discarded canine or third molar extractions or orthodontic exposure, and can be frozen away for many years. Dental follicle stem cells could be a potential cell source for a stem cell bank. In our work we have demonstrated that isolated dental follicle stem cells meet the necessary criteria to be called mesenchymal stem cells and that they have a remarkable osteogenic and mineralization potential, improved by collagen substrate or hydroxyapatite coating of titanium implants. Our research demonstrated that dental follicle stem cells are an excellent source of cells for inducing bone regeneration on dental titanium implants.

## Methods

### Harvesting the dental follicle

Dental follicles were surgically removed from the completely intrabone impacted third molar/canine. The follicular sac was harvested using two different procedures, first during molar odontectomy and second during orthodontical disclosure of the canine.

Odontectomy surgeries respective/canine disclosures for orthodontic purposes were performed by the same oral surgeon OL with 10 years experience.

Subjects included in our research were females with age comprised in the range of 18–40 years. Patients that were included in the study had total intra-osseous molar or canine inclusion. All patients were free of infectious complications, cysts or tumors that may be associated with impacted teeth. The patients included in the study showed no comorbidities. All patients included in our study were handed and ask to read and sign an informed consent approved by the Ethical Committee of the University of Medicine and Pharmacy Iuliu Hatieganu Cluj Napoca. The informed consent described the surgical procedure of odontectomy or orthodontic disclosure of impacted molars and canins. Patients signed that they agree with the surgical procedure of odontectomy or orthodontic disclosure with follicle sac harvesting. The participants gave their written consent.

The clinical protocol was approved by The Ethical Committee of the University of Medicine and Pharmacy Iuliu Hatieganu, Cluj-Napoca, registration number 292/6.05.2011.

### Establishment of cell cultures from the dental follicle

Fragments from dental follicles were placed in 50 ml Falcon tubes containing 15 ml complete medium (DMEM/F-12 10 % fetal calf serum (FCS), 1 % antibiotics, 1 % L-Glutamine, 1 % non-essential aminoacids (NEA). Another fragment was fixed in 10 % formaldehyde for histological studies. Dental follicles fragments were cut in small pieces and washed with PBS (Phosphate Buffer Saline-Sigma). An enzymatic digestive cocktail was used: 0.1 % colagenase IV (Gibco) + 0.25 % trypsin EDTA (Sigma). After 30 min, tissue fragments were washed with complete DMEM/F-12 medium containing 10 % FCS (Fetal Calf Serum). Cell suspensions were filtered with 70 μm Filcons mesh. After centrifugation, obtained monocellular suspension and explants were cultured in Dulbecco’s modified Eagle’s medium (DMEM) high glucose/F-12HAM (Sigma) containing 15 % fetal calf serum (FCS, Sigma), 2 mM L-Glutamine, 1 % antibiotics, 1 % non-essential aminoacids (NEA), 55 μM beta-mercaptoethanol, 1 mM natrium piruvate (all reagents from Sigma), in 25-cm^2^ culture flasks (Nunc) in a humidified 5 % CO2 atmosphere. After 24 h the first adhered cells appeared and after 3 days, when cell monolayers were confluent, the cells were replated by trypsinization, at a 1:3 split ratio, and recultured. The medium was changed at 2–3 days. Cells showed a fibroblastoid shape.

### Analytical methods

#### Histological analysis

Harvested tissues were fixed in 10 % formaldehyde. Tissues were then dehydrated in an ascending series of ethanol and embedded in paraffin. Serial sections of 5 μm were cut in different planes for orientation and staining purposes. Selected sections were stained with hematoxylin –eosin (HE).

#### Phenotypical characterization

Isolated stem cells were characterized through *immunocytochemistry* staining with monoclonal antibodies. 2 × 10^5^ cells at forth passage were seeded on Lab-Tek Permanox chamber slides for 3 days and were fixed with 4 % paraformaldehyde solution in PBS. Cells were permeabilized with 0.1 % Triton X-100 in PBS for 20 min at room temperature. Unspecific antibody bound was blocked with 10 % BSA (bovine serum albumin) in PBS, 20 min at room temperature. For identification of stem cell markers primary monoclonal antibodies were used as follows: CD 34 FITC (Fluorescein isothiocyanate), CD 44 FITC, (purchase from Becton Dickinson, dilution 1:20), CD49 PE (Phycoerythrine), CD90 FITC, CD 117 PE, CD 105 PE, SSEA-4, Oct ¾ (Santa Cruz Biotechnologies, dilution 1;50), and Nanog (R&D Systems). Samples were incubated with primary antibodies 60 min at 4 °C in dark, step followed by three washes with PBS. Secondary antibody IgG FITC from Santa Cruz Biotechnologies was used in the same dilution 1:50 with an incubation period of 45 min at room temperature. Finally, the slides were mounted with DAPI (4,6-diamidino-2-phenylindole) mounting medium (Santa Cruz Biotechnologies) and samples were visualized with a Zeiss Axiovert microscope using filters at 488, 546 and 340/360 nm. Image acquisition was performed with an AxioCam MRC camera.

Stem cells markers expression was investigated also by *flowcytometry*. Briefly, 10 × 10^5^cell per sample were immunolabeled for 60 min at 4 °C with CD 34 FITC, CD44 FITC, CD45 FITC (Becton Dickinson, dilution 1:20) CD73 PE, CD 105 PE, CD29PE,CD49e PE, CD 166 PE and CD117 PE (Santa Cruz Biotechnologies, dilution 1:50). For negative controls, cells were incubated with IgG1 FITC and IgG1 PE (Sigma-Aldrich). After 3 washes with cold PBS, cells were analyzed with BD FACS Canto II, 6-color flowcytometer, using BD FACSDiva version 6.1.3 software.

#### RT-PCR analysis

Expanded cells at 3th passage were analyzed for gene expression: Oct-3/4, Rex-1, TERT, Nanog, Sox-2, SCF, HLA-DRα, Vimentin, c-kit (CD117), Thy-1, (CD90), CBFβ, Tie-2, HLA-ABC, CXCR4.

##### RNA extraction and RT-PCR

Total RNA was isolated from two samples of dental follicle mesenchymal cells (namely DF1 and DF2) in culture (80 % confluence) and from amniotic membrane mesenchymal stromal cells. These placental cells have already been extensively investigated and they were used as a comparative control of mRNA expression. RNA extraction was performed using TRIzol Reagent (Invitrogen) according to the manufacturer’s instructions. One microgram of total RNA was used for reverse transcription with the ImProm Reverse Transcription System (Promega). Only mRNA was transformed into cDNA by using oligo-dT primers in the reaction mixture, together with: AMV reverse-transcriptase 15 u/μg; buffer solution (10 mM Tris–HCl, pH = 9.0; 50 mM KCl; 0.1 % Triton X-100); dNTP solution, 1 mM each; MgCl2 5 mM; recombinant ribonuclease inhibitor 1 u/μl; ultrapure nuclease-free water. The described mixture was incubated at 45 °C for 45 min, followed by heating at 95 °C for 5 min and cooling at 0–5 °C for another 5 min. Subsequently, the cDNA obtained was stored at −20 °C until its use in PCR amplification reactions. Amplification was performed under standard conditions, using the components of the GoTaq PCR Core System II kit (Promega) on a Techne TC3000 thermal cycler (Bibby Scientific Ltd). The cDNA amount was generally 5–10 ng/μL and negative controls were always prepared (ultrapure water instead of standard DNA). The reaction also included: Taq polymerase 0.025 u/μL; buffer solution; dNTP solution 0.2 mM each; MgCl2 1.5 mM; primers and nuclease-free ultra pure water. The program used was a standard amplification scheme in which the melting temperatures of specific primers varied: denaturation 95 °C, 2 min.; 35–45 cycles of 95 °C, 30 s. – t°C (depending on primers), 1 min – 72 °C, 2 min; 72 °C, 5 min; storage at 4 °C. The primer sequences and the size of the amplification product are indicated in the table below (Table 1). The PCR products were then separated by electrophoresis on 2 % agarose gel and photographed with a UV transilluminator. As a loading control for RNA, we used the ubiquitous transcription factor CBFβ.

**Table 1:** The genes, primer sequences and the size of the amplification product

Genes	Primer sequences (5′ → 3′) forward and reverse	Size of the product [bp]
Oct-3/4	aggagtcccaggacatcaaag; tcgtttggctgaataccttc	146
Rex-1	atggctatgtgtgctatgagc; ctcaacttcctagtgcatcc	447
TERT	catcatcaaaccccagaacac; caaacagcttgttctccatgt	371
Nanog	attataaatctagagactccag; tcctgaataagcagatccatg	407
Sox-2	aagcgctttttttgatcctgattc; accacaccatgaaggcattcatg	363
SCF	tatttaatcctctcgtcaaaac; agaattcttcaggagtaaagag	369
HLA-DRα	ttgaagaatttggacgatttg; aaactcccagtgcttgagaagag	407
Vimentin	ttcagagagaggaagccgaaaac; tttaagggcatccacttcacag	422
c-kit (CD117)	ttcagcgagagttaatgattctg; tgtattcacataaacattaaatg	400
Thy-1 (CD90)	aaagaagcacgtgctctttggc; actcagagaagtaggatctctg	379
CBFβ	aacgaggagttttttaggaagc; attcagaatcatgggagccttc	273
Tie-2	tctgtactgcttgtatgaacaatg; tttaccactgtttacttctatatg	405
HLA-ABC	acccaggacacggagctcgtggagacca; cacactttacaagctgtgagagacaca	354
CXCR4	attcctttgcctcttttgcagatata; atggccaggtagcggtccagactgatgaa	418

#### Inducing differentiation into osteogenic lineages

A differentiation protocol using basal and complex osteogenic differentiation medium was performed at 5th passage. 1 × 10^3^ cells/cm^2^ were seeded in 60 mm culture Petri dishes or Lab-Tek Permanox chamber slides for induction of osteogenic differentiation in vitro. Cells were cultured until they reached approximately 80 % confluence, before adding osteogenic differentiation medium. Simple osteogenic medium consisted of DMEM high glucose/F-12(1:1), supplemented with 10 % FCS, 2 mM L-glutamine, 10 nM dexamethasone, 1 % NEA, 50 μg ascorbic acid and 10 mM β glycerol-phosphate (all from Sigma Aldrich). Complex osteogenic medium (OC) contained in addition to OS medium 3 ng/ml Bone Morphogenic Protein -2 (BMP-2) and 2 ng/ml Tranforming Growth Factor β1(TGF-β1) (Table 2).

**Table 2:** Formula used for simple and complex osteogenic mediums

Simple osteogenic differentiation medium (OS)	Complex osteogenic differentiation medium (OC)
• DMEM high glucose:F12 HAM(1:1) • 10 % fetal calf serum • Penicillin(100U/ml) + Streptomycin (100 μg/ml) • 2 mM L-glutamine • Non-essential aminoacids 1 % • 10 mM β glicero-phosphate • 50 μg ascorbic acid • 10 nM dexamethasone	• DMEM glucose :F12 HAM(1:1) • 10 % fetal calf serum • Penicillin(100U/ml) + Streptomycin (100 μg/ml) • 2 mM L-glutamine • Non-essential aminoacids 1 % • 10 mM β glicero-phosphate • 50 μg ascorbic acid • 10 nM dexamethasone • 3 ng/ml BMP2 • 2 ng/ml TGF β1

*Immunocytochemical staining* was performed after 3 weeks of cultivation as described in section Phenotypical characterization of Methods. For demonstration of osteogenic differentiation, primary antibodies were used to evaluate the expression of specific osteoblasts proteins: osteopontin (OP), osteonectin (ON), osteocalcin (OC) and alkaline phosphatase (ALP) (Santa Cruz Biotechnologies). After binding with correspondent secondary goat anti-mouse antibodies, IgG1 marked with FITC (Santa Cruz Biotechnologies), the samples were counterstained with an antifade medium containing DAPI in order to evidence the nuclei and were examined using a Zeiss Axiovert microscope by reversed phase fluorescence using filters at 488 and 340/360 nm. Image acquisition was performed with an AxioCam MRC camera.

#### Cultivation on titanium implants

As a clinical application of DF stem cells in implantology, a biocompatibility study was performed by culturing cells on titanium samples. Three types of titanium scaffolds were used: porous titanium (Ti control) fabricated from Ti6Al7Nb alloy with 25 % total porosity, processed with Selective Laser Melting (SLM) technology), porous titanium infiltrated with hydroxyapatite (TiHA) and with silicatitanate (TiSiO_2_) by a sol–gel method as shown by Brie et al. [42] Medium porosity of the implants infiltrated with HA and SiO2-TiO2 composite was 8 and 12 %, respectively, the distribution of these values being very much dependent on the viscosity of the used sol-gels. After performing these treatments we obtained HA and TiO2 nanocrystals in anatase form, both stable and bioactive. For proving that by used sol–gel method, we obtain these nanocrystalline forms of HA and anatase, after heat treatments at quite low temperatures, as shown in Additional file 6: Figure S6 and Additional file 7: Figure S7.

Titanium samples were design as discs with 10 × 5 × 3 mm sizes. 1.2 × 10^5^ cells/well in 1.5 ml medium were seeded in 12-wells plates onto implants surface in standard stem cell medium. These experiments aimed to evaluate DF stem cells adhesion and proliferation onto titanium implants using a viability test (FDA assay) as well as the osteogenic differentiation potential of cells cultivated on titanium implants’ surface.

##### FDA viability assay

Cells viability and proliferation is an important indicator of biocompatibility of studied materials. Fluorescein diacetate (FDA) test, as an indicator of cells’ metabolic activity, membrane integrity and cells size, was used to evaluate the adhesion, viability and proliferation of DF stem cells cultivated on titanium implants surface. After 1 and 48 h the cells were washed three times with PBS supplemented with Ca^2+^ and Mg ^2+^ and incubated 5 min in dark at 37 °C with 1 ml/well with FDA solution (at a final concentration of 2.4 μM in PBS with Ca ^2+^ and Mg ^2+^). After incubation, the wells were washed twice with PBS and fluorescence intensity (FI) was measured at 488 nm using a BioTek Synergy 2 fluorescence microplate reader, using area scan instrument option. All experiments were performed in triplicate. The same staining method of viable cells was performed after 7 days and the samples were visualized in fluorescence at 488 nm with a Zeiss Axiovert microscope.

#### Osteogenic differentiation of DF stem cells cultivated onto titanium implants

##### ELISA assays

*BMP2* levels were measured using a R&D Quantikine ELISA kit according to the manufacturer’s instructions. DF stem cells were seeded on the surface of titanium implants (Ti control, Ti HA and Ti SiO2) in 12 well plates, at a concentration of 3 × 10^5^ cells in 1.5 ml medium/well in standard stem cells medium, simple osteogenic (OS) and complex osteogenic (OC) differentiation medium. After 8, 14 and 21 days of cultivation, the medium was harvested and analyzed. In each well of a precoated microplate with monoclonal antibody against BMP-2, were added 50 μl of standards and undiluted samples and incubated for 2 h on a shaker at room temperature. Following a wash to remove the unbound protein, 200 μl of anti-BMP-2 conjugate with horseradish peroxidase were added, followed by 2 h incubation and further washing. After 30 min incubation with the substrate solution, the stop solution was added and optical density was determined with a microplate Biotek Synergy2 reader (Synergy HT; BioTek, Winooski, USA) set to 450 nm. We used duplicate for each sample. The optical density values were interpolated from standard curve. From values of each titanium sample were extracted the values corresponding to control medium. Because OC medium contains BMP-2, the values obtained from OC medium controls (without cells) were also extracted from the obtained results from probes of DF stem cells cultivated in presence of OC medium on titanium implants.

*Osteopontin (OPN)* was evaluated in cell culture medium, using an Abbexa ELISA kit according to the manufacturer’s instructions. Briefly, complete medium was harvested at days 8, 14 and 21. In each well of a microplate (coated with mouse monoclonal antibody against OP), 100 μl of standards and undiluted samples were added. After 1.5 h of incubation at room temperature and washing, 200 μl of OPN conjugate were added, followed by another 1.5 h incubation and further washing. After 30 min incubation with the substrate solution, the stop solution was added and optical density was determined with a microplate Biotek Synergy2 reader set to 450 nm.

##### Calcium quantification by Alizarin red assay

The stage of bone development was investigated with Alizarin Red staining, highlighting the extracellular matrix mineralization, in the supernatants of DF cells cultivated on the three types of titanium implants in standard stem cells medium OS and OC medium. The harvested supernatants were centrifugated and washed three times with deionized water at 10,500 rpm 15 min. On the top of the pellets was added 50 μl of alizarin red solution and incubated 5 min. Calcium deposits on titanium implants seeded with DF stem cells were also investigated by same method. Fixed samples with 4 % paraformaldehyde were washed three times with deionized water and incubated 5 min with 2 % Alizarin red solution in deionized water pH 4.1 followed by extensive washing with deionized water and one washing with PBS. The staining is able to identify calcium in tissue sections and cell cultures, based on the reaction between calcium and Alizarin Red S in a Ca^2+^ chelating complex process. A quantification method was carried using a destaining method with 10 % (*w*/*v*) cetylpyridinium chloride (CPC) (Sigma) in 10 mM sodium phosphate (pH 7.0), adding 1 ml of CPC solution/well. After 15 min at room temperature and shaking, 10 μl from the extracted stain were transferred to a 96-well plate and diluted 10 folds with CPC solution. The violet colored supernatant was read with Biotek Synergy 2 microplate reader at 555 nm. The experiments were performed in triplicate. Control values obtained from control samples of Ti implants without cells were extracted from values corresponding to implants samples seeded with cells. The calcium deposition on implants’ surface and in supernatants was quantified by this method after 21 days of cultivation in standard stem cells medium, OS and OC differentiation medium.

##### Scanning electron microscopy (SEM)

After 21 days of cultivation of DF stem cells on Ti Ctrl, Ti HA and Ti SiO2 implants surface in standard stem cell medium, OS and OC medium, the samples were fixed with 4 % paraformaldehyde in PBS, followed by three washes with PBS. Before analysis the samples were immersed in deionized water. Specimens were characterized with a Quanta 3D FEG Scanning Electron Microscope equipped with an energy-dispersive X-ray microanalyzer (EDX). Control implants without cells were also used for SEM and EDX analysis.

##### Immunocytochemical staining of DF stem cells cultivated on titanium implants

To study the potential of DF stem cells to differentiate preferentially in osteogenic progenitors, when the cells were positioned in direct contact with titanium implants, 12 × 10^5^ stem cells were seeded in 12 well-plates containing titanium implants, in 1.5 ml/well of standard cell culture medium. Immunostaining protocol described at section Methods Phenotypical characterization was applied for immunocytochemical staining of DF stem cells after 70 days of cultivation on the three types of implants (TiCtrl, TiHA, TiSiO_2_) in presence of standard stem cell medium expression of alkaline phosphatase (ALP) (conjugated with FITC), collagen 1A (FITC) and osteopontin (Texas red). Samples were counterstained with an antifade medium containing DAPI and were examined using a Zeiss Axiovert microscope by reversed phase fluorescence using filters at 488, 546 and 340/360 nm. Image acquisition was performed with an AxioCam MRC camera.

#### Statistical analysis

Statistical analysis was performed using a GraphPad Prism5.0 software (Graphpad, La Jolla,Ca, USA) for calcium detection by alizarin red method and for ELISA tests. Data were processed with two-way ANOVA and Bonferroni post-test, with setting of *p* value at <0.001. All results are shown as mean ± SD. For FDA assay we used one-way ANOVA analysis of variance and Bonferroni Multiple Comparison Test (*p* value < 0.001).

## Additional files

Additional file 1: Figure S5. Immunocytochemical staining of DF stem cells cultivated with in standard stem cell medium. DF stem cells were cultivated in standard stem cell medium for 3 weeks. Immunocytochemical staining was performed to evaluate the expression of specific osteoblasts protein osteopontin (OP) and alkaline phosphatase (ALP) as described in section Phenotypical characterization of Methods. The samples were counterstained with an antifade medium containing DAPI in order to evidence the nuclei and were examined using a Zeiss Axiovert microscope by reversed phase fluorescence using filters at 488 and 340/360 nm. Image acquisition was performed with an AxioCam MRC camera. In Figure S5 are illustrated the immunostaining results. A weak expression is observed in these images for ALP and negative staining for OP. Figure S5 Fluorescence images of immunocytochemical staining of DF stem cells cultivated for 3 weeks with standard stem cell medium for osteopontin (OP) FITC (A, magnification × 200) and for alkaline phosphatase (ALP) FITC (B magnification × 100). (PNG 235 kb) Additional file 2: Figure S2. FDA (fluorescein diacetate) viability test. DF stem cell adhesion after 1 h as well the proliferation rate during 48 h and 7 days of cultivation on titanium implants surfaces were investigated using FDA assay. Images were captured in fluorescence microscopy at 488 nm with a Zeiss Axiovert microscope. Image acquisition was performed with an AxioCam MRC camera. Figure S2: Fluorescence images captured after FDA staining of DF stemm cells after 1, 48 h and 7 days of cultivation in standard stem cell medium. (Legend: TiCtrl- Ti6Al7Nb alloy porous titanium, TiHA-titanium infiltrated with hydroxyapatite, TiSiO_2_-titanium infiltrated with silicatitanate) (magnification ×100). (PNG 940 kb) Additional file 3: Figure S3. Alamar Blue viability assay. For testing the viability and proliferation rate of DF stem cells cultivated on titanium implants, cells seeded at a cell density of 1.2 × 105 cells/well in 12-wells plates were stained with Alamar blue solution at different periods of time (24 h, 4 and 12 days). Briefly, 100 μl of Alamar blue solution (Invitrogen) was added in each well containing 900 μl stem cell medium or differentiation medium (OS and OC). Each sample was evaluated in triplicate. After 1 h of incubation in dark at 37 °C, the medium was transferred to another 12-well plates and the absorbance was read using a BioTek Synergy 2 plate reader at 570 nm (Winooski, VT, USA). Statistical analysis was performed using *t* test and two-way ANOVA, Bonferroni posttest. Results: No important differences were observed between titanium implants in terms of cell viability. Statistical differences were noticed only for the 24 h culture between cell cultured on control titanium implants (Ti ctrl) and implants infiltrated with HA (Ti HA) (Figure S3). Two-way ANOVA statistical analysis revealed differences regarding the time factor (24 h vs. 12 days and 4 vs. 12 days) Figure S3: Graphical aspect of optical density values (absorbance at 570 nm) of Alamar blue staining of DF stem cells cultivated with standard stem cell medium evaluated after 24 h, 4 and 12 days (Legend: TiCtrl- Ti6Al7Nb alloy porous titanium, TiHA-titanium infiltrated with hydroxyapatite, TiSiO2-titanium infiltrated with silicatitanate). (PNG 1002 kb) Additional file 4: Figure S4. DF stem cells cultivated in stem cell medium and osteogenic differentiation medium OS and OC showed that the type of implant do not influence the cell viability and proliferation. Inducing the osteogenic differentiation caused as expected, the decreasing of cell number after 4 and 12 days of cultivation by comparison with DF stem cells cultivated with stem cells medium but with no statistical differences. Both viability assays highlight the mitochondrial and metabolic activity of the cells, but also the tests could be a measurement of cells’ proliferation index. FDA by virtue of its bipolar side chains passively penetrate the cell membrane and is then cleaved non-specifically by intracellular esterases produced by respiratory activity of cells, which convert non-fluorescent FDA to fluorescein. Metabolically active cells with intact membrane can convert also resaurin (the main component of Alamar blue) to resorufin product that can be measured colorimetric or fluorimetric with a microplate reader. Figure S4: Graphical aspect of optical density values (absorbance at 570 nm) of Alamar blue staining of DF stem cells cultivated onto titanium implants in presence of stem cell medium (m stem), simple osteogenic medium (mOS) and complex osteogenic medium (mOC) evaluated after 4 and 12 days (Legend: TiCtrl- Ti6Al7Nb alloy porous titanium, TiHA-titanium infiltrated with hydroxyapatite, TiSiO_2_-titanium infiltrated with silicatitanate). (PNG 1067 kb) Additional file 5: Figure S1. Neuronal differentiation of Df stem cells. Protocol of neuronal differentiation. DF stem cells were seeded in 6 well plates at cell density of 20 × 10^5^ cells/well. When cells reached confluence a two steps protocol was applied: cells were cultivated for 48 h in presence of neuronal differentiation medium 1 consisting of DMEM high glucose/F12-HAM (1:1 ratio), 10 % fetal bovine serum (FBS), 1 % antibiotics,2 mM glutamine, 1 % non-essential aminoacids (NEA), supplemented with 10 ng/ml Epidermal Growth Factor (EGF), 10 ng/ml basic Fibroblast Growth Factor (bFGF), 2 % B27 and 1 % N2 Supplement. Afterwards cells were exposed to the differentiation medium 2 for 3 weeks: DMEM high glucose/F12-HAM, 10 % FBS, 1 % antibiotics, 2 mM glutamine, 1 % NEA, 1 % N2 Supplement, 2 % B27 Supplement, 3 μM all-trans retinoic acid, 0.5 mM 3-isobutyl-1-methyl-xanthine (IBMX) (all reagents were purchased from Sigma Aldrich). At the end of 3 weeks of DF stem cultivation with neuronal differentiation medium, cells were fixed and immunocytochemical stained for neurofilaments and CD 133 expression. As shown in Figure S1, the stained cells expressed positivity only for neurofilaments. Figure S1: Fluorescence image of DF stem cells induced to differentiate into neuronal cells. Cells were stained with anti neurofilaments antibody conjugated with FITC green), anti CD 133 conjugated with Texas red (red) and nuclei were counterstained with DAPI (magnification ×100). (PNG 451 kb) Additional file 6: Figure S6. The physical chemical characterization of titanium coatings. For proving that by used sol–gel method we obtain these nanocrystalline forms of HA and anatase, after heat treatments at quite low temperatures. Figure S6: XRD pattern of hydroxyapatite sample after 600 ^o^C heat treatment. (PNG 7 kb) Additional file 7: Figure S7. XRD pattern of silicatitanate sample after 400 ^o^C heat treatment. (PNG 6 kb)

## Abbreviations

ALP: Alkaline phosphatase
ASCs: Adipose-derived stromal/stem cells
BMP-2: Bone Morphogenic Protein-2
bFGF: Basic fibroblast growth factor
CD: Cluster of differentiation
Coll: Collagen type 1 alpha
CPC: Cetylpyridinium chloride
DAPI: 4,6-diamidino-2-phenylindole
DF: Dental follicle
DLX3: Distal-less homeobox 3
DMEM: Dulbecco’s modified Eagle’s medium
ECM: Extracellular matrix
EGR 1: The transcription factor early growth response gene 1
FDA: Fluorescein diacetate
FBS: Fetal bovine serum
FITC: Fluorescein isothiocyanate
HGF: Hepatocyte growth factor
IGF1: Insulin-like growth factor1
IL-6: Interleukin 6
IgG1: Immunoglobulin G1
MSC: Mesenchymal stem cells
Nanog: Homeobox transcription protein
NEA: Non-essential aminoacids
Notch1: Transcription protein
Oct3/4: Octamer-binding transcription factor 4
OC: Osteocalcin
ON: Osteonectin
OP: Osteopontin
PBS: Phosphate buffered saline
PE: Phycoerythrin
Rex-1: Reduced expression-1
RT-PCR: Revers transcriptase -polymerase chain reaction
RUNX-2: Runt-related transcription factor 2
SCF: SCF, kit-ligand
SF: Serum-free
SLM: Selective Laser Melting
Sox-2: Sex-determining region Y box 2
SSEA-4: Stage-specific embryonic antigen-4
STRO-1: Stromal precursor antigen-1
TERT: Telomerase reverse transcriptase
Ti Ctrl: Porous Ti6Al7Nb alloy
TiHA: Porous titanium infiltrated with hydroxyapatite
TiSiO_2_: Porous titanium infiltrated silicatitanate
TGF β1: Transforming Growth Factor β1
VEGF: Vascular endothelial growth factor
ZBTB 16: Zinc Finger And BTB Domain Containing 16
