# Supplementary figures and images for: Dental follicle stem cells in bone regeneration on titanium implants
Source: BMC Biotechnol. 2015 Dec 30;15:114. doi: 10.1186/s12896-015-0229-6 (PMC4697321; doi:10.1186/s12896-015-0229-6)

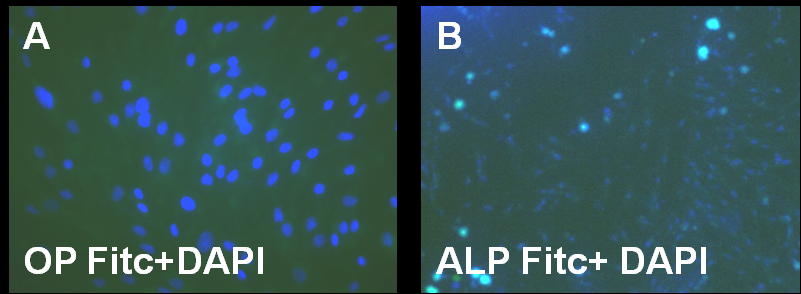

Supplement: Additional file 1: Figure S5. — Immunocytochemical staining of DF stem cells cultivated with in standard stem cell medium. DF stem cells were cultivated in standard stem cell medium for 3 weeks. Immunocytochemical staining was performed to evaluate the expression of specific osteoblasts protein osteopontin (OP) and alkaline phosphatase (ALP) as described in section Phenotypical characterization of Methods. The samples were counterstained with an antifade medium containing DAPI in order to evidence the nuclei and were examined using a Zeiss Axiovert microscope by reversed phase fluorescence using filters at 488 and 340/360 nm. Image acquisition was performed with an AxioCam MRC camera. In Figure S5 are illustrated the immunostaining results. A weak expression is observed in these images for ALP and negative staining for OP. Figure S5 Fluorescence images of immunocytochemical staining of DF stem cells cultivated for 3 weeks with standard stem cell medium for osteopontin (OP) FITC (A, magnification × 200) and for alkaline phosphatase (ALP) FITC (B magnification × 100). (PNG 235 kb) [file 12896_2015_229_MOESM1_ESM.png]

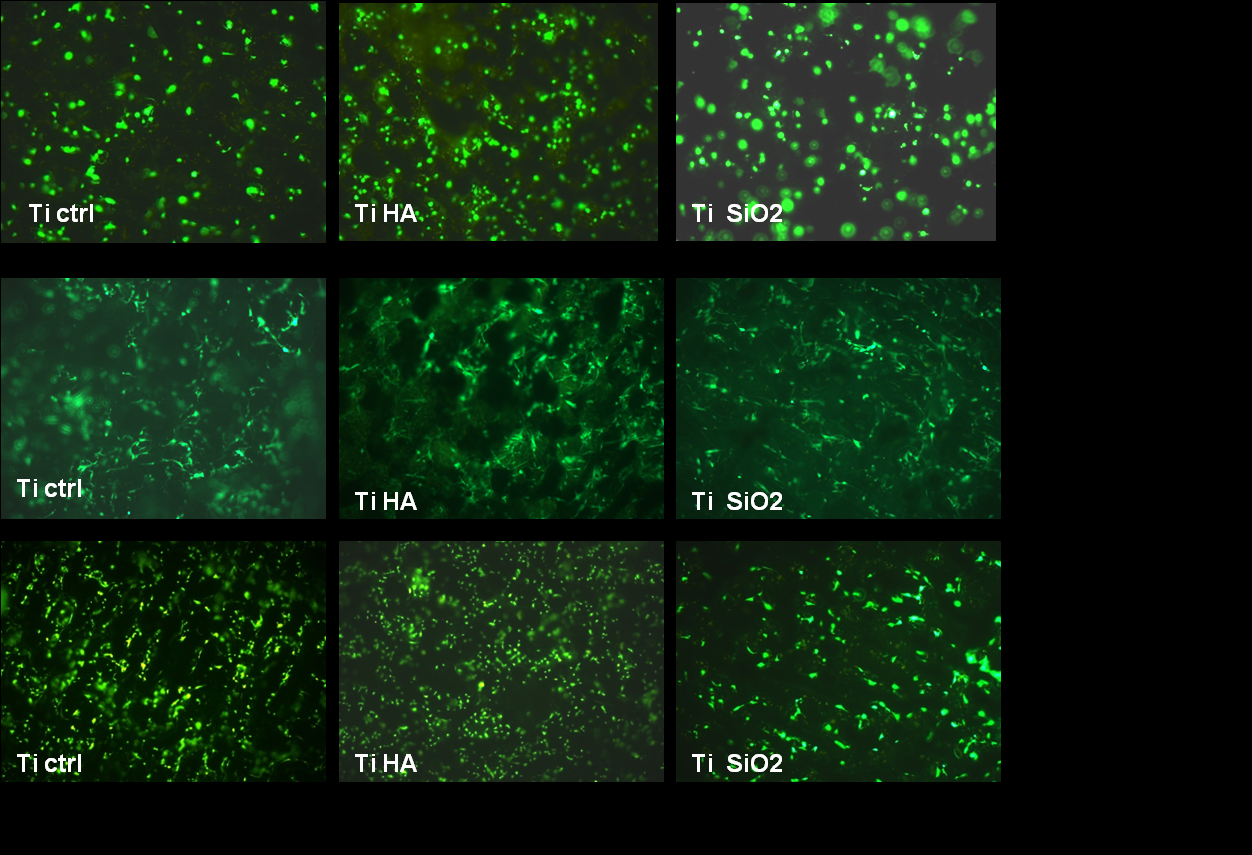

Supplement: Additional file 2: Figure S2. — FDA (fluorescein diacetate) viability test. DF stem cell adhesion after 1 h as well the proliferation rate during 48 h and 7 days of cultivation on titanium implants surfaces were investigated using FDA assay. Images were captured in fluorescence microscopy at 488 nm with a Zeiss Axiovert microscope. Image acquisition was performed with an AxioCam MRC camera. Figure S2: Fluorescence images captured after FDA staining of DF stemm cells after 1, 48 h and 7 days of cultivation in standard stem cell medium. (Legend: TiCtrl- Ti6Al7Nb alloy porous titanium, TiHA-titanium infiltrated with hydroxyapatite, TiSiO2-titanium infiltrated with silicatitanate) (magnification ×100). (PNG 940 kb) [file 12896_2015_229_MOESM2_ESM.png]

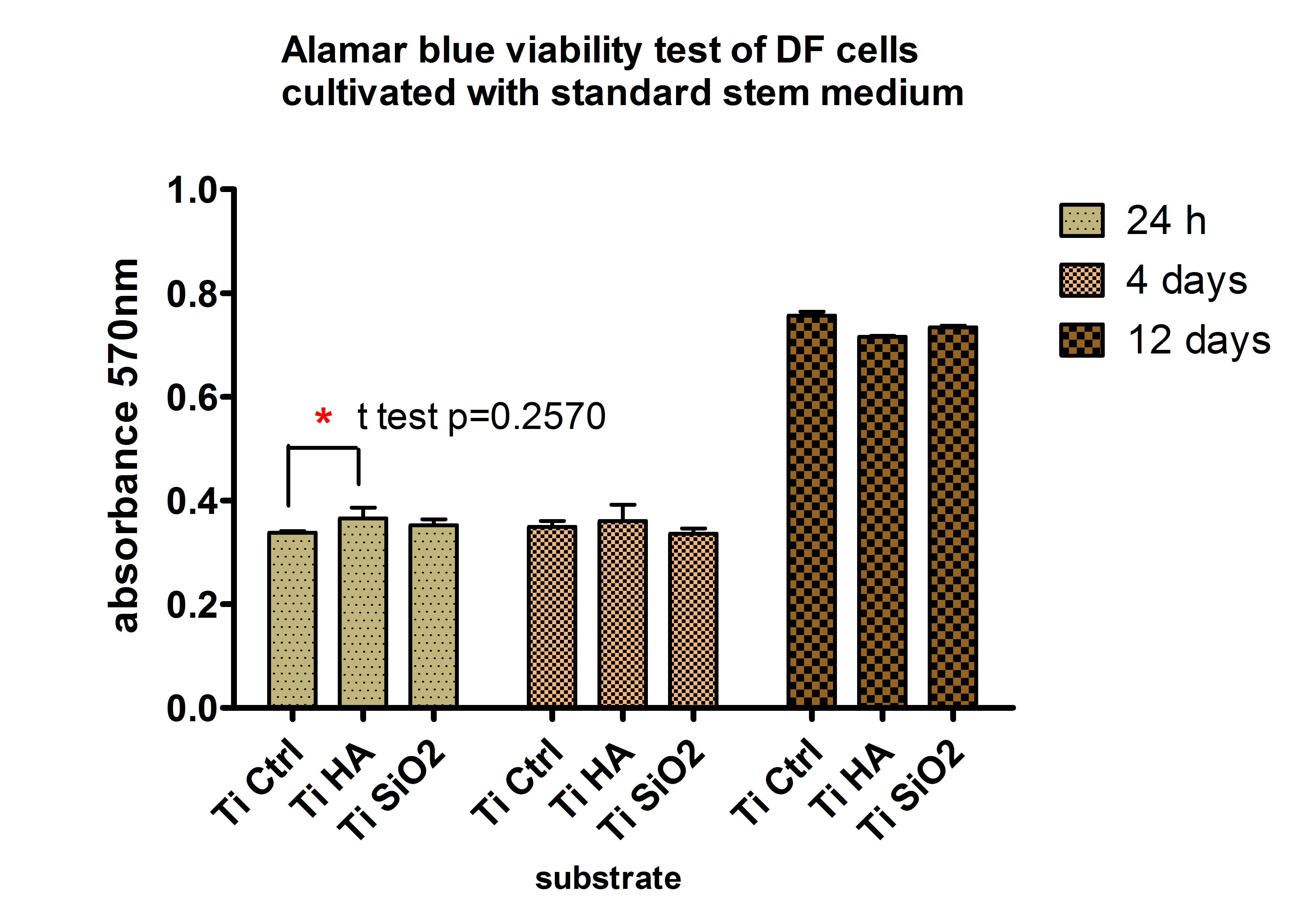

Supplement: Additional file 3: Figure S3. — Alamar Blue viability assay. For testing the viability and proliferation rate of DF stem cells cultivated on titanium implants, cells seeded at a cell density of 1.2 × 10^5^ cells/well in 12-wells plates were stained with Alamar blue solution at different periods of time (24 h, 4 and 12 days). Briefly, 100 μl of Alamar blue solution (Invitrogen) was added in each well containing 900 μl stem cell medium or differentiation medium (OS and OC). Each sample was evaluated in triplicate. After 1 h of incubation in dark at 37 °C, the medium was transferred to another 12-well plates and the absorbance was read using a BioTek Synergy 2 plate reader at 570 nm (Winooski, VT, USA). Statistical analysis was performed using t test and two-way ANOVA, Bonferroni posttest. Results: No important differences were observed between titanium implants in terms of cell viability. Statistical differences were noticed only for the 24 h culture between cell cultured on control titanium implants (Ti ctrl) and implants infiltrated with HA (Ti HA) (Figure S3). Two-way ANOVA statistical analysis revealed differences regarding the time factor (24 h vs. 12 days and 4 vs. 12 days) Figure S3: Graphical aspect of optical density values (absorbance at 570 nm) of Alamar blue staining of DF stem cells cultivated with standard stem cell medium evaluated after 24 h, 4 and 12 days (Legend: TiCtrl- Ti6Al7Nb alloy porous titanium, TiHA-titanium infiltrated with hydroxyapatite, TiSiO2-titanium infiltrated with silicatitanate). (PNG 1002 kb) [file 12896_2015_229_MOESM3_ESM.png]

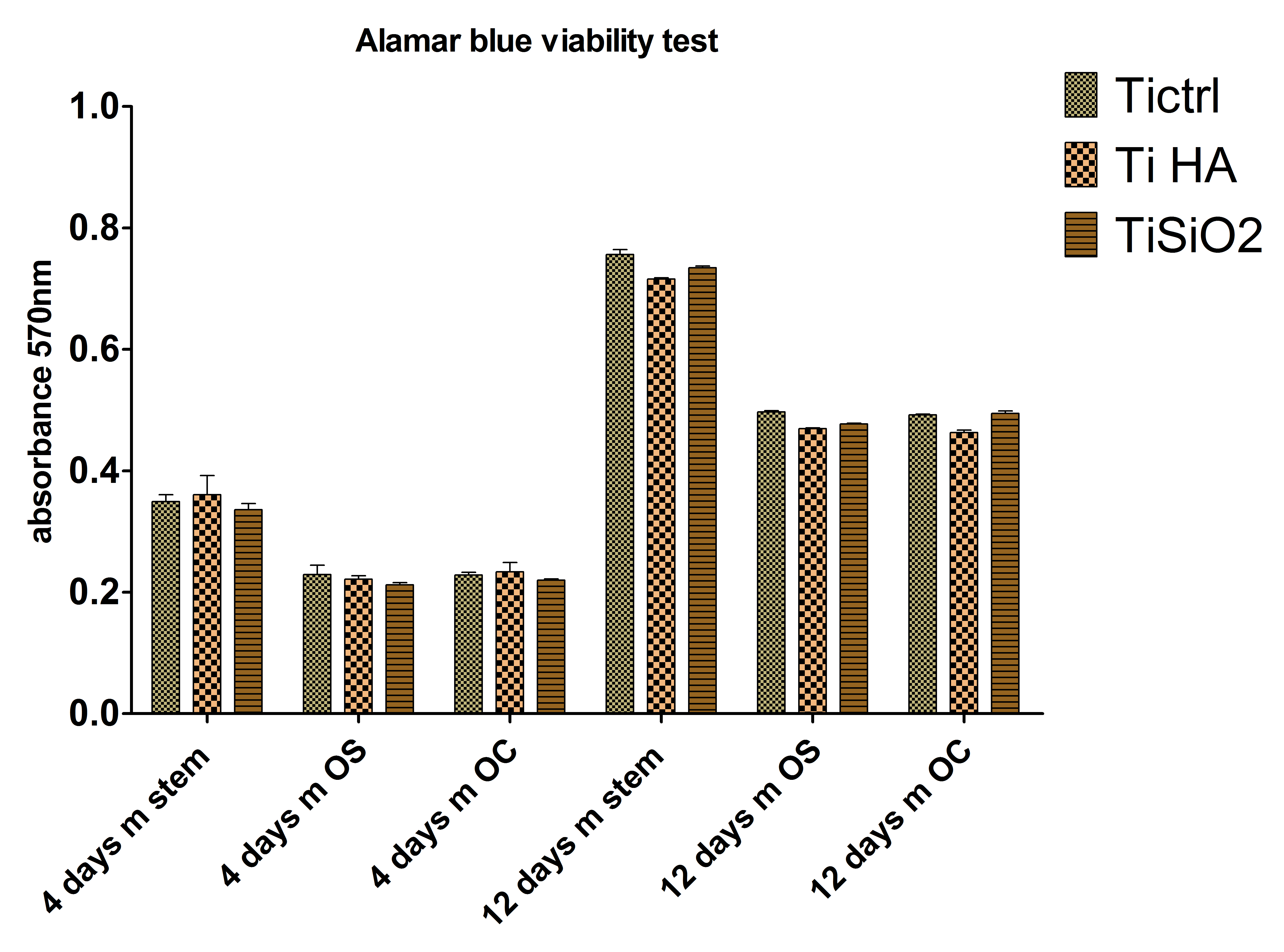

Supplement: Additional file 4: Figure S4. — DF stem cells cultivated in stem cell medium and osteogenic differentiation medium OS and OC showed that the type of implant do not influence the cell viability and proliferation. Inducing the osteogenic differentiation caused as expected, the decreasing of cell number after 4 and 12 days of cultivation by comparison with DF stem cells cultivated with stem cells medium but with no statistical differences. Both viability assays highlight the mitochondrial and metabolic activity of the cells, but also the tests could be a measurement of cells’ proliferation index. FDA by virtue of its bipolar side chains passively penetrate the cell membrane and is then cleaved non-specifically by intracellular esterases produced by respiratory activity of cells, which convert non-fluorescent FDA to fluorescein. Metabolically active cells with intact membrane can convert also resaurin (the main component of Alamar blue) to resorufin product that can be measured colorimetric or fluorimetric with a microplate reader. Figure S4: Graphical aspect of optical density values (absorbance at 570 nm) of Alamar blue staining of DF stem cells cultivated onto titanium implants in presence of stem cell medium (m stem), simple osteogenic medium (mOS) and complex osteogenic medium (mOC) evaluated after 4 and 12 days (Legend: TiCtrl- Ti6Al7Nb alloy porous titanium, TiHA-titanium infiltrated with hydroxyapatite, TiSiO2-titanium infiltrated with silicatitanate). (PNG 1067 kb) [file 12896_2015_229_MOESM4_ESM.png]

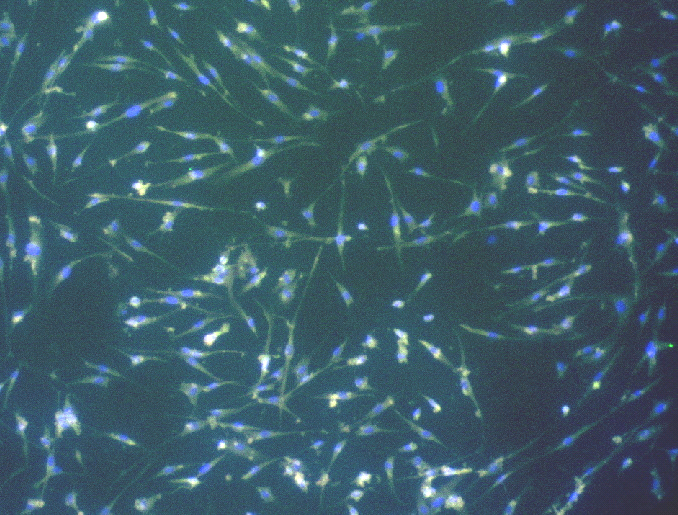

Supplement: Additional file 5: Figure S1. — Neuronal differentiation of Df stem cells. Protocol of neuronal differentiation. DF stem cells were seeded in 6 well plates at cell density of 20 × 105 cells/well. When cells reached confluence a two steps protocol was applied: cells were cultivated for 48 h in presence of neuronal differentiation medium 1 consisting of DMEM high glucose/F12-HAM (1:1 ratio), 10 % fetal bovine serum (FBS), 1 % antibiotics,2 mM glutamine, 1 % non-essential aminoacids (NEA), supplemented with 10 ng/ml Epidermal Growth Factor (EGF), 10 ng/ml basic Fibroblast Growth Factor (bFGF), 2 % B27 and 1 % N2 Supplement. Afterwards cells were exposed to the differentiation medium 2 for 3 weeks: DMEM high glucose/F12-HAM, 10 % FBS, 1 % antibiotics, 2 mM glutamine, 1 % NEA, 1 % N2 Supplement, 2 % B27 Supplement, 3 μM all-trans retinoic acid, 0.5 mM 3-isobutyl-1-methyl-xanthine (IBMX) (all reagents were purchased from Sigma Aldrich). At the end of 3 weeks of DF stem cultivation with neuronal differentiation medium, cells were fixed and immunocytochemical stained for neurofilaments and CD 133 expression. As shown in Figure S1, the stained cells expressed positivity only for neurofilaments. Figure S1: Fluorescence image of DF stem cells induced to differentiate into neuronal cells. Cells were stained with anti neurofilaments antibody conjugated with FITC green), anti CD 133 conjugated with Texas red (red) and nuclei were counterstained with DAPI (magnification ×100). (PNG 451 kb) [file 12896_2015_229_MOESM5_ESM.png]

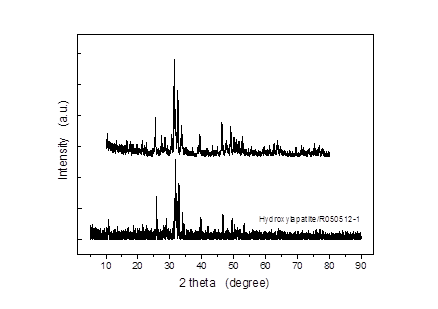

Supplement: Additional file 6: Figure S6. — The physical chemical characterization of titanium coatings. For proving that by used sol–gel method we obtain these nanocrystalline forms of HA and anatase, after heat treatments at quite low temperatures. Figure S6: XRD pattern of hydroxyapatite sample after 600 oC heat treatment. (PNG 7 kb) [file 12896_2015_229_MOESM6_ESM.png]

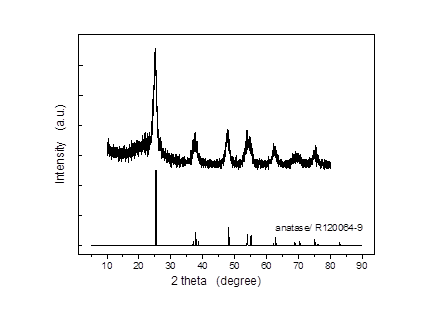

Supplement: Additional file 7: Figure S7. — XRD pattern of silicatitanate sample after 400 oC heat treatment. (PNG 6 kb) [file 12896_2015_229_MOESM7_ESM.png]
